# Supplementary material for: Wealth-related inequality in vitamin A rich food consumption among children of age 6–23 months in Ethiopia; Wagstaff decomposition of the 2019 mini-DHS data
Source: PLoS One. 2024 Oct 8;19(10):e0302368. doi: 10.1371/journal.pone.0302368 (PMC11460695; doi:10.1371/journal.pone.0302368)
Supplement: Attachment — Submitted filename: Response to Reviewers_2.pdf [file pone.0302368.s004.pdf]

Date: 2nd of Jan 2024

Rebuttal letter

Ref: PONE-D-23-16225R1

Title: Wealth-related inequality in vitamin A rich food consumption among children of age 6-23 months in Ethiopia; Wagstaff decomposition of the 2019 mini-DHS data

To: PLOS ONE

Dear all,

We the authors of this manuscript are pleased to thank the journal editors and the reviewers for revising the manuscript and giving your valuable and constructive comments and suggestions that help to improve the manuscript. We have made a rigorous revision of the manuscript as per your questions and comments. We have included the point by point response in the table below framed as reviewers' comment/question and authors' response. The detailed revision and changes we made in the main document are prepared with track changes in the document attached separately. We expect that the revision we made will enable the manuscript to fit the journal. We are happy to receive additional revision if any that would have merit in improving the manuscript.

Editor and Reviewer comments	Authors Response
**Reviewer #1 comments**	
**2nd revision**<br>The authors made revisions, but some points/ revisions are not satisfactory. So, I suggest the authors to look at the following points before the publication of the manuscript<br>1. The gap in the literature on the topic could not be the only reason to justify the importance of the research. Therefore, the justification is still not satisfactory.	**Thank you very much dear reviewer for your comment**<br>We included the justification/rationle for counducting the current study throughout writing the introduction e.g. **lines 90-96**<br>Also, we added a justification statement in the revised manuscript. **Lines 100-102**
2. Under the operationalization section, citation is required, for the variables, at the end of the sentence.	**Thank you again**<br>We have used common citation, particularly the DHS guide, for defining the variables operationally. This has already been cited at the beginning and we opt to keep this for the sake of not making redendecy by citing at the end of each staements. However, we cited a specific literatures to some variables as required. **Page 7**
3. Authors still do not understand the difference between media exposure, and frequency of listening, watching, and reading of Radio, TV, and newspaper respectively. Many researchers have used the radio and TV to measure media exposure and literature, published based on 2019 mini demographic and health survey, shows that media exposure has records in 2019 mini demographic health survey.	**Thank againg for raising this issue.**<br>Ofcourse, some researchers may consider media exposure solely by aggregating the presence of television/radio in the household. However, the mere presence of these does not actually measure the media exposure/use. The correct approach to measure the media exposure is by aggregating the frequency of watching television, listening radio, reading newspapers. These data are however not collected in the 2019 mini EDHS. Theferore, we prefer to keep our analysis as such and

	acknoweledege as limitation about this. **Lines 320--323**
**Reviewer #2 commnets**	**Author Response**
Short title: - written as "Inequalities in iron rich food consumption" which is not related with your main topic. Omit Iron from here.	**Thank you again dear reviewer**<br>Sure, we corrected it and replaced it by vitamin A.
**Abstract:-**<br>1. ''Foods rich in vitamin A (VA) is the only source since it cannot be made in the human body''. The only source of what? This statement does not give any sense and unclear words. Make it clear and specific.<br>Introduction:	**Thank you again dear reviewer for your comment**<br>We have made clarification to the statement in the revised manuscript as follows;<br>Vitamin A (VA) cannot be made in the human body and thus foods rich in VA are the only sources of vitamin A for the body.
2. Lack of flow of idea and redundant ones…..Check Paragraph 3, line 7 up to 11.	**Thank you again**<br>Based on your comment we have paraphrased the paragraph in the revised manuscript. **Lines 70-75**
**Methods and Materials**<br>**Data sources and populations**<br>1. Even though the topic is quite different from previous study done in Ethiopia, ''spatial distribution of vitamin A rich foods intake and associated factors among children aged 6–23 months in Ethiopia: spatial and multilevel analysis of 2019 Ethiopian mini demographic and health survey'', However Data sources and populations some similarities. Make it different from previous studies that were published elsewhere.	**Thank you again**<br>The data source and the population used for the current study was from the mini EDHS 2019 data which is the publicly available at (**https://www.dhsprogram.com**. Due to the publicly accessibility nature of the data and the same methodology and population discreption guiline outlined in the DHS, there could be some similarities across different articles done using this data. However, we have tried to paraphrase and make different to the previous studies.
**Variables of the study:**<br>2. You interviewed the child taken fish any time in the last 24 hours preceding the interview and considered as good consumption of foods rich in	**Thank you very much dear reviwer for your comment**<br>Indeed, the gold standard method to deal with the level of vitamins and minerals in the body is by

vitamin A coded as “1”, otherwise, no consumption coded as “0”. So, could you believe that such like judgment is scientifically sound? Since your data classify based on 24 hours consumption only.	measuruing the blood level of the nutrients or by using some dietetric assessment approaches. However, the present study is based on the secondary survey data on large population where the other dietary assessment methods would not be feasible. For this reason, the data collection method was based on the 24-hour food recall questionnaire, the commonly used method in national dietary survey studies (Caswell et al., 2015, Salvador Castell et al., 2015).<br>We actually, acknowledge for the presence of the possible recall bias in the limitation section of the manuscript. **Lines 320-323**
3.1. How do you know that this child was taken fish/VAC always or not?	**Thank you again**<br>Though it is as stated in the previous response, it is assumed that the 24-hour food recall assessment could reflect the usual household feeding practice.
3.2. How do you know if this child was only taken fish for the first and last at those times?	**Thank you again**<br>Same to the above
3.3. Assume that this child was consumed VAC/fish always, but probably did not take any fish/VAC within 24 hours, what was your judgment?	**Thank you dear reviewer**<br>Similarly, this is actually a survey data that countries worldwide used to assess health staus, health indicators, and dietary patterns at population level using the most feasible approach every five years. The assessment is thought to represent the average usual nutritional habit of the household. And we the researchers have no control over the data collection method in such reglar national surveys. We however produce evidences based on the available data by interpreting the findings

	cautiously and by taking in account all the possible limitations.
3.4. What about the other outcome variables listed 1-6?	**Thank you dear reviewer**<br>The outcome varaiable, consumption foods rich in vitamin A, was assessed through the 24-hour food recall assessment questionnaire as described in the previous reseponses.
4. Add operational definitions	**Thank you again**<br>We have included the operational definations. **Page 7**
**Data management and analysis**<br>1. ‘’Its value ranges from − 1 and + 1, where C = 0 shows perfect equality while C < 0 indicates VAC is disproportionately concentrated among the poor (pro-poor inequality, and C>0 means VAC is disproportionately concentrated among the poor (pro-rich inequality)’’. From this interpretation both C<0 & C>0 indicates it was almost similar i.e. ‘’VAC is disproportionately concentrated among the poor (pro-poor inequality’’. Why you need to state as different interpretation? Again how it was similar? Check it again and make correction.	**Thank you dear reviewer for asking this**<br>That was a typo-error in which the word “poor” would not have been used when C was greater than 0. We made correction in the revised manuscript where the word **poor** was replaced by **rich** and interpreted as follows;<br>Its value ranges from − 1 and + 1, where C = 0 shows perfect equality while C < 0 indicates VAC is disproportionately concentrated among the poor (pro-poor inequality), and C>0 means VAC is disproportionately concentrated among the rich (pro-rich inequality) (Wagstaff et al., 2007).
**Results**	
1. Description of Vitamin A consumption by the maternal and child characteristics:-You put table 1 caption but no table is found at the place. It’s better if you put the description and its table together	**Thank you dear reviewer**<br>There is table 1 and its located at the end of the manuscript **(page 20)** as per the format of the Plos One manuscript writng guideline.<br>https://journals.plos.org/plosone/s/file?id=wjVg/PLOSOne_formatting_sample_main_body.pdf

2. Decomposition of wealth-related inequality in VAC:- You put table 2 caption but no table is found at the place. It's better if you put the description and its table together.	**Thank you again dear reviewer**<br>Same to the above, table 2 its located at the end of the manuscript **(page 21)** as per the format of the Plos One manuscript writng guideline.
**Discussion**<br>1. Some of variables were not discussed well and some doesn't have adequate justification.	**Thank you dear reviewer**<br>We made a revision to the variables and made some more ellborated discussion points to the variables and added possible and plausible justification points as well. **Page 13**. We welcome further comments specific to certain variables, if any so that we can do more.
2. Again at the end your discussion you put single line i.e. ''In this study, only 39%'' statements which is not clear and not discussed. Make it clear.	**Thank you dear reviewer**<br>We were unable to find a text ''In this study, only 39%'' in the discussion. We might have corrected it previously. We are still happy to receive if any.
**Conclusion**<br>1. Conclusion at end was not related with the conclusion you stated in the Abstract section (Region is contributing factor for VAC)….check it again.	**Thank you dear reviewer**<br>We have revised for the consistency of the conclusion statements in the abstract section and at the end of the manuscript and included the variable region.
2. Where is your recommendation?	**Thank you dear reviewer**<br>We have already included the recommendation as follows;<br>The finding calls for the need to multi-sectorial approaches to address the underlying contributing factors to the inequality in VAC identified in this study. Intervention strategies should prioritise addressing the significant modifiable factors identified in this study. For instance, efforts to enhance access to ANC and health institutions delivery services is highly recommended for

	delivering health-facility-based nutrition education and interventions in the study setting. **Lines 329-334**
**Ethical consideration**<br>1. Write the Ethical consideration at the beginning of Methods and materials.	**Thank you dear reviewer**<br>As per the Plos One journal guideline, the Ethical consideration should be written at the end of the methods section or immediately before the result section.<br>https://journals.plos.org/plosone/s/submission-guidelines
**List of Tables**<br>1. Table 1 has some discordant i.e. Women's age, Marital status, Household wealth index, ANC Visit and Children Age in month was 1498 but your total study participants was 1497. How it could happen? Please, check it attentively.	**Thank you dear reviewer**<br>Such discordants in the number of observations in each categories of the variables had happened when we made an approximation to the nearest digit. Based on your review, we have re-analysed the tabulation and made correction in the updated version of the manuscript. **Table 1 pages 19-21**
2. Again on your Table 1:-You state one variable as "Highest education". What it mean? Please, change into Mother educational status. Again don't use any abbreviation like<br>U-5, ANC, PNC and others.	**Thank you dear reviewer**<br>Based on your recommentdation, we have replaced the variable **"**Highest education" by mother educational status in the table and other sections of the manuscript. Regarding the abbreviation like U-5, ANC, PNC, we have indicated a foot note under that table to reduce the inside table bulkiness and to ease understanding as;<br>**ANC**-antenatal care, **BF**-breast feeding, **C** – concentration index, **PNC**-postnatal care.
3. Table 2: The topic was about VAC among children aged 6–23 months but it was mixed with mother characteristics like Age, Marital status, Education,	**Thank you dear reviewer**<br>Sure, we have update the caption/table discreption as follows in the revised manuscript.

Residence, etc. So update this table description.	**Table 2**: Decomposition of the concentration index of wealth-related inequalities to VAC attributable to maternal and child factors among children aged 6–23 months in Ethiopia; mini EDHS 2019.
**List of Figures**<br>4. No need to write as list of figures at the end however, it's better if you only put figures with caption.	**Thank you again**<br>We made this to meet the Plos One journal manuscript writing guideline.
5. Labeling and description of Figure 1 was not written well.	**Thank you dear reviewer for commenting this**<br>For better understanding, we have clarified the caption of figure 1 as follows;<br>**Figure 1**: Proportions of the consumption of food items rich in VA by children of age 6-23 months in Ethiopia
	Finaly, we the authors are very grateful to the reviewers for taking your precious time and contributed significantly to the improvement of the manuscript.

Kindest regards!

Mehari Woldemariam Merid, corresponding author

CASWELL, B. L., TALEGAWKAR, S. A., DYER, B., SIAMUSANTU, W., KLEMM, R. D. & PALMER, A. C. 2015. Assessing Child Nutrient Intakes Using a Tablet-Based 24-Hour Recall Tool in Rural Zambia. *Food Nutr Bull,* 36**,** 467-80.

SALVADOR CASTELL, G., SERRA-MAJEM, L. & RIBAS-BARBA, L. 2015. What and how much do we eat? 24-hour dietary recall method. *Nutr Hosp,* 31 Suppl 3**,** 46-8.

WAGSTAFF, A., O'DONNELL, O., VAN DOORSLAER, E. & LINDELOW, M. 2007. *Analyzing health equity using household survey data: a guide to techniques and their implementation*, World Bank Publications.
