# Supplementary material for: Wealth-related inequality in vitamin A rich food consumption among children of age 6–23 months in Ethiopia; Wagstaff decomposition of the 2019 mini-DHS data
Source: PLoS One. 2024 Oct 8;19(10):e0302368. doi: 10.1371/journal.pone.0302368 (PMC11460695; doi:10.1371/journal.pone.0302368)
Supplement: Attachment — Submitted filename: Comments.docx [file pone.0302368.s002.docx]

Comments to the authors

Abstract:

1. The first sentence in the introduction of your abstract is not clear. Vitamin A is the only source for what? The second sentence is not consistence with the first one. Make it clear.
2. In the introduction section of your abstract, avoid abbreviation (VAC), or use the bracket in the objective of your abstract to make it clear.
3. What is the correct term to write Wagstaff, or wag staff. Be consistent in writing.
4. In the result section of the abstract, I did not get the importance of VAC in line 38. Better if you replace it with A.
5. The recommendations in the abstract section are not informative, for instance how stakeholders improve pre-natal and institutional delivery. Specifically, what mechanisms have you recommended to improve prenatal care and institutional delivery?

Introduction:

1. I am not clear when you wrote “Inadequate micro nutrient feeding practice …..are the key determinant….. optimal growth, and development…” line 54. It needs revision
2. The authors lack to show the gap of the problem from global to local in the background section of the manuscript. So, please articulate the gap more
3. Rationally of the study is not well stated.

Method:

1. The outcome variable is not clearly written. Is “infant and young children aged……. who have taked ……..” a variable? I expect the outcome variable as “wealth-related inequality in vitamin A consumption”. Doesn’t it? If not, please write the outcome variable shortly, and then write how you measure it shortly and clearly. Plus, “have taked” is not grammatically correct.
2. From the outcome variable section, Infants and young children aged 6-23 months by themselves could not able to respond whether they have taken vitamin A-rich food or not. So, how do you know whether they take it or not? Make the readers clear about the respondents.
3. Operationalize what occupation, region, use of prenatal, use of antenatal care, current breast-feeding status, birth order, and number of under-five children in the family, family size, and household wealth index mean in your study?
4. Author missed important attributes such as media spots such as television and radio

Result:

1. Item used in figure one is not representative, including other vitamin A-rich food items

Discussion:

Strength and limitation:

1. For me, the topic which is being studied for the first time in some areas may not manifest its strength. Be specific, and write the exact strength of your paper. Avoid citation in this section.

Conclusion and recommendation:

1. The recommendation in this section is copied from the recommendation written in the abstract section. Better if you avoid duplication.
