# Supplementary material for: Wealth-related inequality in vitamin A rich food consumption among children of age 6–23 months in Ethiopia; Wagstaff decomposition of the 2019 mini-DHS data
Source: PLoS One. 2024 Oct 8;19(10):e0302368. doi: 10.1371/journal.pone.0302368 (PMC11460695; doi:10.1371/journal.pone.0302368)
Supplement: Attachment — Submitted filename: Response to Reviewers_1.docx [file pone.0302368.s003.docx]

Date: 05 November, 2023

**Rebuttal letter**

##### Ref: PONE-D-23-16225

## Title: Wealth-related inequality in vitamin A rich food consumption among children of age 6-23 months in Ethiopia; Wagstaff decomposition of the 2019 mini-DHS data

### To: PLOS ONE

**Dear all,**

We the authors of this manuscript are pleased to thank the journal editors and the reviewers for revising the manuscript and giving your valuable and constructive comments and suggestions that help to improve the manuscript. We have made a rigorous revision of the manuscript as per your questions and comments. We have included the point by point response in the table below framed as reviewers’ comment/question and authors’ response. The detailed revision and changes we made in the main document are prepared with track changes in the document attached separately. We expect that the revision we made will enable the manuscript to fit the journal. We are happy to receive additional revision if any that would have merit in improving the manuscript.

| **Editor and Reviewer comments** | **Authors Response** |
| --- | --- |
|  | |
| **Editor comments** | |
| Journal Requirements:  When submitting your revision, we need you to address these additional requirements.  1. Please ensure that your manuscript meets PLOS ONE's style requirements, including those for file naming. The PLOS ONE style templates can be found at  <https://journals.plos.org/plosone/s/file?id=wjVg/PLOSOne_formatting_sample_main_body.pdf> and  <https://journals.plos.org/plosone/s/file?id=ba62/PLOSOne_formatting_sample_title_authors_affiliations.pdf> | Thank you very much dear reviewer for your comments  Based on your suggestions, we re-visited the PLOS ONE submission guidelines and made amends such as section rearrangement (e.g moving the ethics statement to the methods section), renaming some subhedings (previously background now replaced by Introduction), and others in the recvised manuscript accordingly. |
| 2. We noticed you have some minor occurrence of overlapping text with the following previous publication(s), which needs to be addressed:  - <https://doi.org/10.1371/journal.pone.0281681>  - <https://doi.org/10.1186/s40795-022-00521-y>  - <https://doi.org/10.1080/16549716.2022.2040152>  In your revision ensure you cite all your sources (including your own works), and quote or rephrase any duplicated text outside the methods section. Further consideration is dependent on these concerns being addressed. | Thank you again dear editor  Yes, we have noticed some overlapping texts especially in the background section and now we made intense revision and parapharased the each sections of the revised manuscript. Pages 3-5 of the introduction section and other pages of the method section. |
| 3. Your ethics statement should only appear in the Methods section of your manuscript. If your ethics statement is written in any section besides the Methods, please move it to the Methods section and delete it from any other section. Please ensure that your ethics statement is included in your manuscript, as the ethics statement entered into the online submission form will not be published alongside your manuscript. | Thank you again dear editor for your comment  As per your comment and the PLOS ONE guidline, we have moved the ethics statenment to the method section in the revised manuscript. Lines 206-212 |
| 4. Please include captions for your Supporting Information files at the end of your manuscript, and update any in-text citations to match accordingly. Please see our Supporting Information guidelines for more information: <http://journals.plos.org/plosone/s/supporting-information>. | Thank you again  Yes, we have included the captions for all the supporting documents (figures) in the revised manuscript |
| **Reviewer 1** |  |
| Abstract:  1. The first sentence in the introduction of your abstract is not clear. Vitamin A is the only source for what? The second sentence is not consistence with the first one. Make it clear. | **Thank you very much dear reviwer for your comment**  Yes, we made the required revision.  Line xxx |
| 2. In the introduction section of your abstract, avoid abbreviation (VAC), or use the bracket in the objective of your abstract to make it clear. | **Thank you again**  Sure, we made the requested change. **Lines 27-29** |
| 3. What is the correct term to write Wagstaff, or wag staff. Be consistent in writing.. | **Thank you again**  The correct one is Wagstaff and we use this consistently in the revised manuscript. |
| 4. In the result section of the abstract, I did not get the importance of VAC in line 38. Better if you replace it with A | **Thank you dear reviewer**  Sure, that was typo error. We corrected it in the revised manuscript. |
| 5. The recommendations in the abstract section are not informative, for instance how stakeholders improve pre-natal and institutional delivery. Specifically, what mechanisms have you recommended to improve prenatal care and institutional delivery? | **Thank you dear reviewer**  In the revised manuscript, we paraphrased the recommendation as follows;  Nutritional related interventions should prioritise children of poorer households and less educated mothers. Moreover, enhancing access to ANC and health facilities delivery services through education, advocacy, and campaign programs is highly recommended in the study setting. **Lines 50-53** |
| Introduction: 1. I am not clear when you wrote “Inadequate micro nutrient feeding practice …..are the key determinant….. optimal growth, and developent…” line 54. It needs revision | **Thank you dear reviewer for commenting this**  We re-write the statemenmt as follows;  Inadequate micro-nutrients feeding practices to infants and young children, particularly in the first 2 years of life, are the key risk factors for under-nutrition and delayed cognitive development and growth mainly in developing countries [1]. Good VA consumption refers to the intake of foods rich in VA at least one food item among the main type of food items [2]. **Lines 56-59** |
| 2. The authors lack to show the gap of the problem from global to local in the background section of the manuscript. So, please articulate the gap more | **Thank you again**  We have made a severe revision in the background section to highligjht the gap from global to local cointext as per your comment. **Page 3-4 starting line 62** |
| 3. Rationally of the study is not well stated. | Thank you again  In the revised manuscript, we tried to show the rationale of the study clearly.  **Lines 91-100** |
| Method: 1. The outcome variable is not clearly written. Is “infant and young children aged……. who have taked ……..” a variable? I expect the outcome variable as “wealth-related inequality in vitamin A consumption”. Doesn’t it? If not, please write the outcome variable shortly, and then write how you measure it shortly and clearly. Plus, “have taked” is not grammatically correct. | **Thank you dear reviewer for commenting this**  Sure, we corrected the definition of the outcome variable and the grammatical error as follows;  In this study, the outcome varaiable was wealth-related inequality in consumption of foods rich in vitamin A among infants and young children aged 6–23 months living with their mother who have taken at least one food item among the seven food items……. **Lines 121-131** |
| 2. From the outcome variable section, Infants and young children aged 6-23 months by themselves could not able to respond whether they have taken vitamin A-rich food or not. So, how do you know whether they take it or not? Make the readers clear about the respondents. | **Thank you dear reviewer**  Ofcurse, the questions/items themselves tells that the mothers/caregivers were asked to assess weather they fed their inants/children foods rich in VA or not. However, we added the following statements in the revised manuscript for more clarification based on your comment.  ….These questions were asked to the mothers’ or the caregivers’ of the infants any time in the last 24 hours preceding the survey. Lines xxx |
| 3. Operationalize what occupation, region, use of prenatal, use of antenatal care, current breast-feeding status, birth order, and number of under-five children in the family, family size, and household wealth index mean in your study? | **Thank you againg dear reviewer**  We included the operational definations of the variables used in the analysis in the revised manuscript. **Lines 138-158** |
| 4. Author missed important attributes such as media spots such as television and radio | **Thank you again for asking this.**  Sure, media exposure is really important determinant factor for VAC. However, the frequency of listening to radio, watching television, and reading newsletter were not recoreded/available in the mini EDHS 2019 in which media exposure has to be generated. |
| Result:  1. Item used in figure one is not representative, including other vitamin A-rich food items | **Thank you again**  Ofcaurse, there could be other sources of VA. But the DHS guidline/reports and other previous researches consistently considered the seven (egg, meat, carrot, green leafy, mango, organs, fish ) major food items as the main sources of VA. |
| Discussion: Strength and limitation:  1. For me, the topic which is being studied for the first time in some areas may not manifest its strength. Be specific, and write the exact strength of your paper. Avoid citation in this section. | **Many thanks dear reveiewer for your comment**  Baased on your comment, we have amended the strength and limitation statement in the revised manuscript. **Lines 315-319** |
| Conclusion and recommendation:  1. The recommendation in this section is copied from the recommendation written in the abstract section. Better if you avoid duplication. | **Thank again**  We have made the required revision as per your comment. **Lines 325-330** |
| Reviewer #2: Topic was very fantastic and Everything that was stated in the manuscript was also very interesting. How ever, there is some topographical error and some issue not clear for now since your data was derived from secondary data thus why there is minor similarities with studies done before from the same data with different topic. In addition, please follow PLOS ONE Guidelines. | **Many thanks dear reviewer for your comments**  Sure, we have went through line by line and made correction to errors and amends to as required of the revised manuscript. |
|  | *Finaly, we the authors are very grateful to the editors and reviewers for taking your precious time and contributed significantly to the improvement of the manuscript*. |

Kindest regards!

**Mehari Woldemariam Merid, corresponding author**
